# Defining operational research priorities to improve malaria control and elimination in sub-Saharan Africa: results from a country-driven research prioritization setting process

**DOI:** 10.1186/s12936-023-04654-8

**Published:** 2023-07-30

**Authors:** Roger Tine, Samantha Herrera, Mouhamed Ahmed Badji, Kyle Daniels, Pascal Ndiaye, Cara Smith Gueye, Fassiatou Tairou, Laurence Slutsker, Jimee Hwang, Evelyn Ansah, Megan Littrell, The Malaria Operational Research Prioritization Partnership, Abigail Pratt, Alassane Dicko, Baltazar Candrinho, Busiku Hamainza, Cara Smith Gueye, Kyle Daniels, Catherine Maiteki-Sebuguzi, Charles Mbogo, Corine Karema, Core Ngufor, Don Mathanga, Dorothy Achu, Elizabeth Juma, Evelyn Ansah, Fitsum Tadesse, Frank Burkybile, Jenny Carlson, Jaishree Raman, Khoti Gausi, Pascal Ndiaye, Perpetua Uhomoibhi, Richard Steketee, Roopal Patel, Rose Leke

**Affiliations:** 1 Université Cheikh Anta Diop, Dakar, Senegal; 2 PMI Insights Project/PATH, Washington, DC USA; 3 PMI Insights Project/University of California, San Francisco Malaria Elimination Initiative, San Francisco, USA; 4 Mabouya Solutions, Brussels, Belgium; 5 U.S. President’s Malaria Initiative, Malaria Branch, U.S. Centers for Disease Control and Prevention, Atlanta, GA USA; 6 University of Health and Allied Sciences, Accra, Ghana

**Keywords:** Research prioritization, Malaria control, Malaria elimination, Sub-Saharan Africa

## Abstract

**Background:**

In order to reignite gains and accelerate progress toward improved malaria control and elimination, policy, strategy, and operational decisions should be derived from high-quality evidence. The U.S. President’s Malaria Initiative (PMI) Insights project together with the Université Cheikh Anta Diop of Dakar, Senegal, conducted a broad stakeholder consultation process to identify pressing evidence gaps in malaria control and elimination across sub-Saharan Africa (SSA), and developed a priority list of country-driven malaria operational research (OR) and programme evaluation (PE) topics to address these gaps.

**Methods:**

Five key stakeholder groups were engaged in the process: national malaria programmes (NMPs), research institutions in SSA, World Health Organization (WHO) representatives in SSA, international funding agencies, and global technical partners who support malaria programme implementation and research. Stakeholders were engaged through individual or small group interviews and an online survey, and asked about key operational challenges faced by NMPs, pressing evidence gaps in current strategy and implementation guidance, and priority OR and PE questions to address the challenges and gaps.

**Results:**

Altogether, 47 interviews were conducted with 82 individuals, and through the online survey, input was provided by 46 global technical partners. A total of 33 emergent OR and PE topics were identified through the consultation process and were subsequently evaluated and prioritized by an external evaluation committee of experts from NMPs, research institutions, and the WHO. The resulting prioritized OR and PE topics predominantly focused on generating evidence needed to close gaps in intervention coverage, address persistent challenges faced by NMPs in the implementation of core strategic interventions, and inform the effective deployment of new tools.

**Conclusion:**

The prioritized research list is intended to serve as a key resource for informing OR and PE investments, thereby ensuring future investments focus on generating the evidence needed to strengthen national strategies and programme implementation and facilitating a more coordinated and impactful approach to malaria operational research.

**Supplementary Information:**

The online version contains supplementary material available at 10.1186/s12936-023-04654-8.

## Background

Despite significant progress over the past two decades in reducing malaria-related morbidity and mortality, malaria remains an important public health threat, especially in sub-Saharan Africa (SSA) [1]. In 2021, there were an estimated 247 million malaria cases and 619,000 deaths worldwide, with greater than 90% occurring in SSA [1]. Although some countries have continued to make progress, in many the fight against malaria has either slowed, stalled, or in some cases reversed in recent years [1, 2]. Funding for malaria prevention and control has also plateaued since 2015 [3, 4]. Given these trends, it has become increasingly important to revive efforts to accelerate progress so that countries get back on track to achieve malaria mortality and morbidity reduction goals and targets as defined in the World Health Organization (WHO) Global Technical Strategy for Malaria 2016–2030 [5]. To reignite progress, evidence-based guidance is needed on best practices for malaria control and elimination, how to best tailor and target interventions at subnational levels for greatest impact, and how to effectively incorporate new tools into programmes [6–8], particularly in the context of limited resources.

Research prioritization efforts for malaria to date have often been ad hoc. Some countries have processes to define national research priorities for malaria control and elimination, but often the resulting research agenda is not updated regularly, shared broadly, or tracked for progress [9, 10]. Further, there have not been any efforts to synthesize country-defined research priorities that may have broader relevance across multiple country settings [9, 10]. At a global level, previous initiatives like the Malaria Eradication Research Agenda (malERA) in 2011 and the malERA Refresh in 2017, represent robust efforts to prioritize key research topics at a broader level for malaria eradication [8, 11], but these focused predominantly on priorities related to basic sciences and technology and development of new tools [8, 12–17]. Thus, malERA was less relevant for supporting national malaria programmes (NMPs) to address key operational challenges and bottlenecks in the implementation of their programmes and guide evidence-based programming decisions and adaptations at country level.

Current donor and government investments in malaria control and elimination are predominantly focused on delivery of interventions [7]. Operational research (OR) and evaluation are often not prioritized for funding, as they may be viewed as competitive with the purchase of costly essential commodities [7, 18]. Within this context, the U.S. President’s Malaria Initiative (PMI) in partnership with the Bill & Melinda Gates Foundation (BMGF) and the Global Fund to Fight AIDS, Tuberculosis, and Malaria (GFATM), expressed interest in supporting a country-driven research prioritization effort to better understand country-identified research priorities and improve overall coordination and efficiency of OR and programme evaluation (PE) efforts. To do this, PMI funded the PMI Insights project, a multidisciplinary partnership tasked with generating and catalysing the use of OR and PE evidence to inform malaria programme decision-making, to design and implement a country-driven research prioritization process in collaboration with the Université Cheikh Anta Diop (UCAD) in Dakar, Senegal.

UCAD and PMI Insights facilitated a broad stakeholder consultation process to identify pressing operational challenges and critical evidence gaps in malaria control and elimination policy, strategy, and guidelines, and to define a priority list of OR and PE topics to address these challenges and gaps. The overarching objective of this effort was to foster improved alignment of country research priorities with those of key funding agencies to better coordinate and align future malaria OR and PE efforts.

## Methods

The research prioritization process consisted of four main stages that were carried out between February 2021 through February 2022: (1) design of the process (Feb–Jun 2021); (2) synthesis of existing information (Mar–Jun 2021); (3) gathering of stakeholder input on priority research topics (Jul–Oct 2021); and (4) evaluation and ranking of the identified research priorities against a set of defined evaluation criteria (Dec 2021–Feb 2022).

### Stage 1: Design of the process

**Fig. 1:**
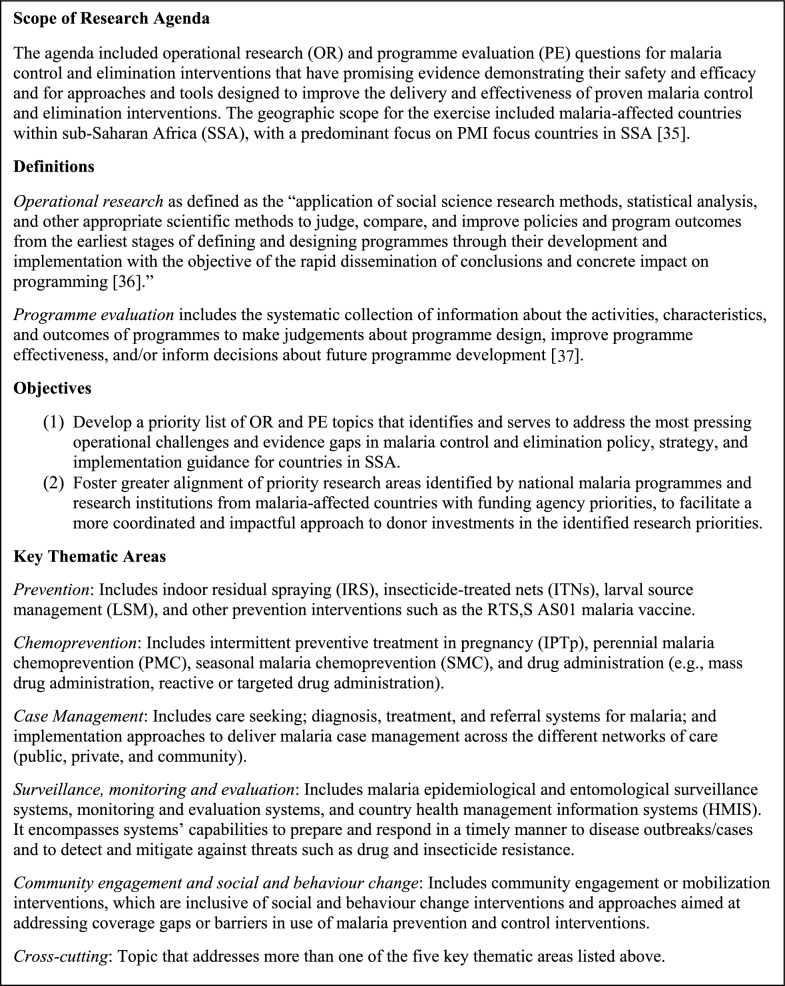
Overview of Malaria OR and PE prioritization setting scope and objectives

An overarching framework was first developed to guide the implementation of the process [19]. The framework defined the scope of the research agenda, the objectives of the process, and the thematic areas for organizing and synthesizing information gathered (Fig. 1). The framework also outlined broadly the approach for implementing the process and the evaluation criteria that would be used for scoring and ranking the identified research priorities (Additional file 1: Table S1). A detailed protocol was subsequently developed to further describe the overall process and tools for gathering input from stakeholders [20]. The protocol was submitted to PATH’s Research Determination Committee, the National Ethics Committee for Health Research in Senegal, and the U.S. Centers for Disease Control and Prevention, and was determined to be non-human subjects’ research.

### Stage 2: Synthesis of existing information

A document review was conducted to capture information on: (1) malaria operational challenges and bottlenecks faced by NMPs within SSA in the implementation of their programmes; (2) evidence gaps in national or global malaria policy, strategy, and implementation guidance; and (3) outputs from recent national, regional (within SSA), and/or global level research prioritization processes. To focus on gathering more current and relevant operational challenges, evidence gaps, and research prioritization outputs, the scope of the review was limited to documents, reports, and literature from 2015 through 2021. The review included current or most recent National Malaria Strategic Plans from PMI focus countries, PMI Malaria Operational Plans from 2019 and 2020, recent WHO Malaria Programme Review and Mid-term Review reports from countries within SSA, WHO Evidence Review Group meeting reports, WHO Global Malaria Programme guideline development group meeting reports, WHO Malaria Policy and Advisory Group meeting reports, Roll Back Malaria Working Group meeting reports, Cochrane reviews of specific malaria interventions, and reports or publications on country, regional, and global-level malaria research prioritization research outputs.

All documents were reviewed, coded, and analysed in the online qualitative software programme Dedoose using the defined thematic areas (Fig. 1). Further details on the methodology used for the document review and the full list of documents included in the review are available in a separate report [21]. The document review findings were triangulated with the findings from the stakeholder consultations in Stage 3 of the process.

### Stage 3: Gathering stakeholder input

To gather stakeholder input, a mixed-methods approach was used that entailed key informant interviews (KIIs), focus group discussions (FGDs), and an online survey with individuals from five target stakeholder groups: NMPs in PMI focal countries within SSA; academic/research institutions from malaria-affected countries in SSA; WHO country and Africa Regional Office representatives; technical partners working in or providing support to malaria research or programming; and funding agency representatives from PMI, the Global Fund, and Bill & Melinda Gates Foundation. A stakeholder mapping was conducted to identify potential participants from these five key stakeholder groups. Partners engaged in the prioritization process, including PMI Insights consortium partners, UCAD, PMI, BMGF, and GFATM, were asked to provide recommendations for individuals to consult. RBM working group member lists and malaria organization and project websites were also reviewed to identify potential participants.

KIIs and FGDs were conducted with all target stakeholder groups, except for technical partners. Participants for KIIs and FGDs were selected purposively based on their role within their organization and experience in malaria control and elimination research and/or programming. Participant sampling was conducted in a way to ensure a diverse group of participants based on experience working in different transmission settings; geographic representation was taken into account for stakeholders based within SSA. Selected participants were invited to participate in an interview via email. For the NMPs and research institutions, the participants contacted were encouraged to invite other representatives from their institution to participate. Representatives from each of the stakeholder groups were selected to participate in either a KII or FGD based on participant availability. For the FGDs, there was no mixing of participants across institutions. Altogether, 15 NMPs, 18 research institutions, and representatives from WHO, PMI, GFATM, and BMGF were targeted for KIIs and FGDs. KIIs and FGDs were conducted using a semi-structured interview guide, and were carried out in English, French, and Portuguese as appropriate. Participants verbally consented to participate prior to the interviews. Interviews were conducted using Microsoft Teams or Zoom virtual platforms.

An online survey was used to gather inputs from technical partners identified through the stakeholder mapping. All identified participants from the stakeholder mapping (151 in total) were sent the online survey using the SurveyMonkey platform. The survey was shared in English and French, and participants were asked for written consent for their participation. The online survey was available for six weeks, with up to 3 reminders sent to the participants.

The three main themes explored in the KIIs, FGDs, and online survey were similar to those investigated in the document review: (1) key operational challenges and bottlenecks experienced by NMPs in the implementation of their programmes; (2) evidence gaps in national and global malaria policy, strategy, and implementation guidance; and (3) priority OR and PE questions that could help to address the key challenges and identified evidence gaps. Data from the KIIs, FGDs, online survey, and the document review were analysed and organized by the key thematic areas in the prioritization framework, the three key topic areas explored (operational challenges/bottlenecks, evidence gaps, and priority OR and PE questions), and across the different stakeholder groups. To identify which OR and PE topics would be selected for the evaluation process (Stage 4), only topics that were identified by at least three stakeholder groups and/or through the document review (at least three different sources) or by at least three NMPs or research institutions, were prioritized for evaluation and ranking.

### Stage 4: Evaluating and ranking the identified research priorities

An independent evaluation committee was formed to conduct the evaluation of the OR and PE priority topics identified during Stage 3. The committee comprised 17 representatives from NMPs (n = 6, one of whom included a former NMCP Director), research institutions within malaria-affected countries (n = 9), and WHO (n = 2). Committee members were selected to ensure diverse representation across geographic areas with SSA, malaria area(s) of technical expertise, gender, and type of institution.

The evaluation process and scoring methodology used was adapted from the Child Health and Nutrition Research Initiative (CHNRI) research priority setting methodology [22, 23]. The six evaluation criteria used for the evaluation were initially defined in Stage 1 (Table 1, Additional file 1: Table S1) and reviewed and agreed upon by the evaluation committee. Evaluation committee members were asked to independently evaluate the identified topics from Stage 3 against the six evaluation criteria. For each evaluation criterion, the evaluator was asked one to two questions to assess whether the identified research topic satisfied the evaluation criteria—a total of ten evaluation questions for each topic (Table 1). Committee members used a five-point Likert scale to score the topic against each evaluation question; however, evaluators were given the option to note “do not know.”

**Table 1:** Evaluation criteria and questions

Evaluation criteria	Evaluation questions
Broad relevance	Q1. Is it likely the research findings could inform policy, strategy, or implementation guidance across several (3 +) malaria-endemic countries?
High impact on malaria burden	Q2. Does the research question address a significant barrier to achieving coverage targets of a proven or new promising malaria control or elimination intervention? Q3. Is it likely the research would enable or lead to a substantial reduction in malaria burden or bring a setting(s) closer to elimination?
Improves efficiency	Q4. Is it likely the research could inform how to optimize the delivery of an intervention in terms of reducing unnecessary costs or resources? Q5. Is it likely the research would inform how to improve the quality or overall effectiveness of an intervention?
Addresses inequities	Q6. Would populations most-at-risk for and/or most vulnerable to malaria likely benefit from the research after the findings have been applied or implemented? Q7. Does answering the research question have the potential to lead to more equitable coverage of interventions or in the disease burden distribution in the mid- or long-term (5–10 years)?
Scalability and sustainability	Q8. Does the research address an intervention or approach that could be feasibly delivered at scale by national malaria programmes?
Feasibility	Q9. Is the research question clear and well framed? Q10. Is it feasible to design and conduct a study in response to the research question (considerations: time and cost to undertake study, human resource needs, study design/methods, would receive ethical approval without major concerns)?

For each OR/PE topic, a research priority score (RPS) and average expert agreement (AEA) score was calculated. The RPS score was calculated by taking the average score across all criteria, for each OR/PE topic. The AEA was calculated as the percent of evaluators who chose the mode for each evaluation criteria question, averaged across the ten evaluation questions (see Additional file 1 for additional details). “Do not know” responses were not included in the calculation of the RPS and AEA scores. OR/PE topics were ranked highest to lowest by their RPS score, with a higher score denoting a higher level of agreement with the evaluation criteria. After evaluation committee members independently evaluated the topics, the committee was convened to review the evaluation scores and provide recommendations for how the OR/PE topics could be reworded for improved clarity.

## Results

In total, 128 malaria experts provided their input through the KIIs, FGDs, and online survey (Table 2). Four NMPs and 12 research institutions did not respond to the invitation to participate in an interview. Of the initially sampled 15 NMPs and 18 malaria-endemic research institutions, four alternate NMPs and five research institutions in SSA were selected as replacements from the initial sample due to non-response. No alternates were required for the participants from the WHO or funding agencies. For the document review, a total of 109 documents were reviewed and synthesized. Forty-six country- and global-level technical experts and researchers gave inputs through the online survey.

**Table 2:** Summary of inputs gathered and synthesized in prioritization process

Source	Summary of inputs gathered
Document review	109 documents reviewed
Interviews (encompasses interviews and focus group discussions (FGDs))	• Interviews with 14 national malaria programmes • Interviews with 11 malaria-endemic research institutions • Interviews with 6 WHO representatives from sub-Saharan Africa • Interviews with staff from 4 funding agencies (PMI (USAID/CDC), BMGF, GF, and NIH) In total: 47 interviews/FGDs were conducted with a total of 82 participants
Online stakeholder survey	46 survey participants
All sources	128 participants

### Pressing operational challenges and evidence gaps

The most salient operational challenges and pressing evidence gaps identified by stakeholders and through the document review are summarized by key thematic areas in Table 3. Many of the operational challenges reported impact all intervention areas and broadly relate to the poor-quality delivery of the intervention or service, lack of or limited access to interventions, and broader health systems deficiencies related to insufficient financial resources available to achieve or sustain high coverage of interventions, supply chain weaknesses, insufficient human resource capacity, limited and poor-quality data for decision-making, and poor linkages with the private sector. Stakeholders also noted the general lack of evidence on cost-effectiveness and effectiveness of several intervention areas and the need for more evidence on best practices or strategies to address the common barriers/operational bottlenecks to delivery of high-quality and coverage of core interventions.

**Table 3:** Commonly reported operational challenges and evidence gaps in malaria prevention and control, by thematic area

Thematic area	Most common operational challenges identified	Key evidence gaps reported
Prevention	ITNs • Sustaining high coverage in high burden areas and ensuring coverage among highest risk populations • Routine distribution and distribution to the last mile • Low ITN use in populations with high access • Shorter than expected durability IRS • How to determine what insecticide to spray, and when/where to deploy IRS LSM • Engagement of communities on carrying out and maintaining LSM • Not enough trained human resources for mapping breeding sites • Lack of M&E frameworks and indicators for LSM; general insufficient monitoring of LSM programmes Crosscutting • Implementation of insecticide resistance management	ITNs • Insufficient evidence on effectiveness of CE/SBC approaches to improve ITN use • Understanding of barriers and facilitators to ITN use (specifically social factors at community level, in the context of provider-patient interactions, and in low transmission settings) • ITN durability under routine conditions IRS • Best practices for IRS withdrawal and transition strategies to prevent case resurgence • Impact of IRS and focal or reactive IRS on malaria burden, transmission, and insecticide resistance • Cost-effective and cost-saving approaches for IRS LSM • Impact of LSM on malaria burden and transmission in different contexts and transmission settings Crosscutting • Effective delivery mechanisms and innovative approaches to reach and ensure sustained coverage in hard-to-reach populations • Effectiveness and cost-effectiveness of vector control intervention combinations • Understanding around the essential data elements needed and at what granularity to inform and improve targeting and stratification of interventions
Chemoprevention	IPT • Delayed first antenatal care (ANC) visit or incomplete attendance at recommended ANC visits • Access to ANC/IPTp services (e.g., transport, cost) • Health care providers low adherence to IPTp guidelines/lack of training on IPTp guidelines SMC • Measurement of coverage due to inaccurate denominators • High cost of implementation	IPT • Effective strategies for achieving high coverage and efficient delivery of IPTp • What transmission threshold to use to inform transition away from IPTp delivery • Factors that have impeded scale-up of perennial malaria chemoprevention (PMC) and strategies for addressing the barriers • Limited understanding of the operational feasibility and best delivery platform for PMC SMC • Effectiveness and cost-effectiveness of SMC, particularly in use among school age children and geographical coverage outside the Sahel • Effective strategies for achieving high coverage of SMC in target areas • When and where to use SMC to reduce burden and when and how to determine when to scale-up or scale-down SMC MDA • Optimum methods for implementing MDA in different settings/contexts
Case management	Overarching • Poor access to health care services • Caregivers’/patients’ perception of poor-quality services (e.g., long wait times, stockouts, insufficient providers) • Health care providers’ poor adherence to national case management guidelines • Challenge with commodity quantification due to poor quality or limited availability of stock data; limited capacity of health facility staff in supply chain management, reporting and use of stock data; and use of parallel sources/mechanisms for quantification and distribution Community case management • High turnover of community health workers (CHWs) and insufficient coverage of CHWs • CHWs’ poor adherence to national treatment guidelines • Poor linkages between communities and CHWs • Weak supervision of CHWs • Poor data quality at community level and lack of integration of community data into national HMIS Private sector case management • Poor or lack of engagement, coordination, and integration between private and public sector for case management and reporting • Poor adherence to national treatment guidelines	Overarching • Effective strategies for improving health care providers’ adherence to national treatment guidelines (beyond training and supervision) • Effective strategies for addressing stockouts Community case management • Evidence on the quality of integrated community case management provision by CHWs Private sector case management • Effective strategies and policies for strengthening collaboration of the private sector in malaria case management and reporting into the national HMIS
Surveillance, monitoring, and evaluation	SME/HMIS • Poor quality HMIS data • Limited capacity in surveillance, monitoring and evaluation/operational research, particularly in data analysis, interpretation and use of data for programmatic decision-making • Poor culture of data use • Inadequate supervision for data reporting and limited or inconsistent administration of data quality audits • Fragmented/poorly integrated data systems • Limited knowledge/capacity around how best to stratify to inform subnational targeting of interventions Entomological monitoring and surveillance • Limited coverage of entomological surveillance data • Limited capacity for conducting entomological. surveillance and for the analysis, interpretation and use of vector data • Fragmented systems for entomological data capture	SME/HMIS • Guidance on minimum data needs to inform real-time programmatic decision-making, particularly for malaria surveillance systems in low transmission settings and for informing subnational targeting of interventions • Identification and characterization of key populations, including accurate denominators for populations at risk to improve accuracy of intervention coverage measurement • Understanding the current performance of surveillance systems, particularly in low transmission settings • Optimal/effective surveillance system approaches for malaria elimination Entomological monitoring and surveillance • Limited evidence on *An. stephensi* spread in new geographical areas, including information on breeding, resting, and biting behaviours, and susceptibility to insecticides
Community engagement/social and behaviour change	• Insufficient monitoring and evaluation of CE/SBC activities • Limited technical capacity in CE/SBC	• Effectiveness and cost-effectiveness of CE/SBC interventions on malaria intervention uptake in different transmission settings and contexts • Evidence on duration of the effectiveness of malaria CE/SBC interventions
Crosscutting	• Insufficient funding to achieve high coverage of interventions • Supply chain delays or failures due to a myriad of challenges • Insufficient number of trained human resources to provide sufficient coverage of health care services, prevention interventions, and SME	• Evidence on effective multi-sectoral strategies for malaria prevention • Evidence on effectiveness and cost-effectiveness of different malaria intervention packages

### Operational research and program evaluation priorities

Altogether, 33 OR and PE topics were identified through the consultation and document review synthesis. The RPS across the 33 identified research topics ranged from 71.5 to 87.9 (out of 100). The AEA ranged from a low of 40.3 to a high of 67.6 (out of 100). Table 4 presents the ranking of the 33 topics by their RPS and includes their overall AEA score (see Additional file 1: Tables S2 and S3 for the detailed scores of the research topics across each evaluation criterion). By key thematic area, ten OR and PE topics were identified for prevention; seven for chemoprevention and case management; five for surveillance, monitoring and evaluation; two for community engagement and social behaviour change; and two were identified as crosscutting thematic areas. Generally, the RPS across the 33 topics did not range substantially, with the top 15 topics receiving a RPS of 80 or above.

**Table 4:** Overall rank of malaria operational research and programme evaluation topics

Rank	Operational research/programme evaluation topic	Thematic area(s)	Research priority score	Average expert agreement score
1	Test and evaluate different delivery mechanisms to reach and sustain high coverage of ITNs among hard-to-reach and highest risk populations	Prevention	87.9	59.4
2	Evaluate the effectiveness and cost-effectiveness of different strategies for deploying the RTS, S AS01 malaria vaccine with chemoprevention (e.g., campaign vs. expanded programme on immunization (EPI)-linked vs combination campaign/EPI strategies)	Prevention and chemoprevention	86.6	53.0
3	Assess the effectiveness and cost-effectiveness of different intervention combinations (e.g., ITNs + IRS, ITNs or IRS + LSM, vector control + chemoprevention) to better understand how interventions should be combined to maximize impact	Crosscutting	85.3	53.5
3	Test and evaluate approaches or interventions to reduce the frequency of stockouts of key commodities for malaria case management, especially at the community level (specifically addressing challenges related to commodity quantification, stock management capacity, reporting and use of stock data)	Case management	85.3	47.9
5	Evaluate and compare different insecticide management and/or rotation strategies on insecticide resistance prevalence and intensity (crosscuts use of ITNs and IRS)	Prevention	85.1	54.1
6	Evaluate the impact and cost-effectiveness of expanding the age range, geographical coverage, and rounds of treatment of seasonal malaria chemoprevention (SMC)	Chemoprevention	84.5	55.3
7	Assess factors associated with volunteer community health worker (CHW) cadres’ motivation and retention and evaluate different approaches or interventions to improve volunteer CHW motivation and retention	Case management	83.3	47.6
8	Assess predictors of adherence to and determinants of uptake of SMC and evaluate different strategies to achieving high SMC coverage and adherence	Chemoprevention	82.3	52.5
9	Test and evaluate the effectiveness of different deployment and targeting approaches for IRS to maximize impact (e.g., testing different insecticides, duration and frequency of spraying, geographic/structural targeting strategies)	Prevention	82.0	50.6
10	Assess different approaches or interventions to improve the analytic and data use capacity, and data use culture at different levels of the health system	SME	81.3	45.3
10	Assess the impact of IRS and focal/reactive IRS on malaria burden, transmission, and insecticide resistance	Prevention	81.3	52.4
10	Given the challenges with ITN durability, test and evaluate the effectiveness of different approaches to improve routine/continuous distribution channels for ITNs to sustain coverage between mass campaigns	Prevention	81.3	60.4
13	Compare different CE/SBC strategies in terms of effectiveness and cost-effectiveness on healthcare seeking, adherence to treatment, and uptake of key prevention interventions	CE/SBC	80.9	55.1
14	Assess the effectiveness and cost-effectiveness of innovative approaches to reduce the cost and/or improve the efficiency of IRS implementation (e.g., partial spraying of structures, use of a decentralized approach, targeted spraying)	Prevention	80.8	55.9
15	Assess structural and behavioural factors associated with delayed care-seeking across different population groups (e.g., age, gender, hard-to-reach/vulnerable populations) and compare different strategies to decrease delays in care-seeking	Case management	80.0	54.7
16	Assess predictors of adherence and non-adherence to case management treatment guidelines among health care providers and test/evaluate different strategies to improve adherence to guidelines	Case management	79.5	46.1
17	Evaluate how current surveillance systems are functioning, and whether they are producing reliable and accurate information to guide countries toward elimination	SME	79.4	48.2
18	Assess the operational feasibility and most effective delivery platform for perennial malaria chemoprevention administration (e.g., EPI, mass campaign, community health workers)	Chemoprevention	78.9	42.8
19	Assess the feasibility and benefit of different digital tools/systems for use at the community level for data capture, reporting, and transmission to HMIS/DHIS2	SME	78.7	45.3
20	Evaluate different strategies for achieving high MDA coverage and adherence in different transmission contexts	Chemoprevention	78.6	47.1
20	Test and evaluate interventions to improve adherence to malaria treatment guidelines and reporting in the private sector (Note: Private sector is inclusive of private sector clinics, hospitals, pharmacies, drug shops, and other private sector providers)	Case management	78.6	55.3
22	Assess the long-term effectiveness and sustainability of different SBC approaches on key malaria treatment and prevention behaviours and the duration of their impact on intervention uptake	CE/SBC	78.1	60.9
23	Compare different strategies for surveillance and response in elimination settings, assessing completeness, timeliness, delivery of response, and cost-effectiveness	SME	78.0	55.6
23	Test the effectiveness of different strategies to improve IPTp coverage	Chemoprevention	78.0	58.2
25	Test and evaluate strategies to improve the efficiency of the delivery of IPTp (e.g., community-based delivery through community health workers)	Chemoprevention	77.9	46.8
26	Test and evaluate different approaches or interventions for improving HMIS data quality (e.g., assess minimum periodicity of supervision, strategies for easing reporting burden on staff/simplification of reporting system, strategies to incentivize reporting accuracy)	SME	77.6	57.1
27	Evaluate different strategies to improve health care worker adherence to integrated management of childhood illness guidelines	Case management	77.4	58.1
28	Evaluate the effectiveness and cost-effectiveness of LSM on epidemiological and entomological outcomes in different transmission contexts and the duration of impact	Prevention	76.6	52.4
28	Test approaches or strategies to improve cost and resource efficiency (e.g., integration of seasonal malaria chemoprevention with other delivery platforms) and to maintain effectiveness in the delivery of SMC when scaling up the intervention	Chemoprevention	76.6	67.6
28	Compare or evaluate different strategies/packages of interventions to prevent resurgence of malaria cases following the withdrawal of IRS	Prevention	76.6	47.1
31	Assess barriers and facilitators to ITN use in different settings where access to ITNs is high and evaluate the effectiveness of different SBC approaches/interventions to improve ITN use within different settings/contexts based on the identified barriers (e.g., community level strategies, provider/patient communication/SBC approaches, SBC approaches for low transmission settings)	Prevention and CE/SBC	76.3	40.3
32	Test different approaches for working with/incentivizing participation and collaboration of the private sector in the referral, diagnosis, treatment, and reporting of malaria cases	Case management	75.5	53.5
33	Assess the impact of cross border movement of people on malaria incidence/prevalence and evaluate the effectiveness of different strategies to reduce malaria transmission across international borders	Crosscutting	71.5	45.0

The top three ranked OR and PE priorities were in the areas of prevention and chemoprevention, with the highest ranked topic focusing on testing and evaluating different delivery mechanisms to reach and sustain high coverage of insecticide-treated nets (ITNs) among hard-to-reach and highest risk populations. The second and third ranked topics related to generating evidence on the effectiveness and cost-effectiveness of different combinations of prevention and chemoprevention interventions, including addressing the combination of the new RTS, S/AS01 malaria vaccine with chemoprevention.

Several prevention topics addressed indoor residual spraying (IRS), including testing and evaluating approaches for maximizing IRS impact (topic #9), assessing the impact of IRS and focal/reactive IRS (topic #10), assessing approaches for reducing the cost/improving the efficiency of IRS (topic #14), and evaluating different strategies or packages of interventions to prevent the resurgence of malaria cases following the withdrawal of IRS (topic #28). Topics related to ITNs included evaluating approaches to improve routine/continuous distribution channels (topic #10) and evaluating the effectiveness of social and behaviour change (SBC) approaches/interventions to improve ITN use in settings where access to ITNs is high (topic #31). A crosscutting prevention topic on evaluating different insecticide management or rotation strategies on insecticide prevalence and intensity ranked fifth.

Of the topics on chemoprevention, three addressed seasonal malaria chemoprevention (SMC)—evaluating the impact and cost-effectiveness of expanding the age range, geographical coverage, and rounds of treatment of SMC (topic #6); evaluating strategies for achieving high SMC coverage and adherence (topic #8); and testing of approaches to improve cost and resource efficiency in the delivery of SMC (topic #28). The two topics on intermittent preventive treatment in pregnancy (IPTp) had similar focuses—evaluating strategies to improve IPTp coverage (topic #23) and efficiency of the delivery of IPTp (topic #25). The last topic addressed assessing the most effective delivery platform for perennial malaria chemoprevention (PMC) in infants (topic #18).

Seven topics addressed the area of case management, which encompassed case management in public and private sectors, as well as at community level. The highest ranked topic in case management was on testing and evaluating approaches or interventions to reduce the frequency of stockouts of key commodities for malaria case management, with a focus on community-level stockouts (topic #3). Other case management topics addressed community health workers’ motivation and retention (topic #7), delayed care-seeking (topic #15), and improving health care provider adherence to malaria case management and integrated management of childhood illness guidelines (topics #16, #20, and #27). The lowest ranked case management topic was on testing different approaches for working with or incentivizing the participation of the private sector in the referral, diagnosis, treatment, and reporting of malaria cases (topic #32).

The top ranked topics in surveillance, monitoring, and evaluation (SME) included assessing approaches for improving analytic and data use capacity, and data use culture (topic #10), evaluating the current performance of surveillance systems (topic #17), and assessing the feasibility and benefit of different digital tools/systems for use at the community level for data capture, reporting, and transmission to HMIS/DHIS2 (topic #19).

In terms of cross-cutting topics, two of the prioritized topics related to SBC/community engagement (CE) focused on generating evidence on the effectiveness and cost-effectiveness of different SBC/CE approaches or interventions on healthcare seeking, adherence to treatment and uptake of key prevention interventions (topic #13), and assessing the long-term effectiveness and duration of impact of different SBC approaches for malaria treatment and prevention behaviours (topic #22). The last crosscutting topic (topic #33) focused on assessing the impact of cross-border movement of people on the malaria burden and evaluating the effectiveness of different strategies to reduce malaria transmission across international borders.

## Discussion

The findings from the research prioritization process highlighted the most pressing challenges and critical evidence gaps facing NMPs in SSA and identified a set of OR and PE priorities that are directly linked to finding solutions and filling the gaps for many of them. The 33 priority topics identified in this process all received a relatively high RPS (above 70), demonstrating that the evaluation committee generally felt all topics were important to address. The lower and more wide-ranging AEA scores across the topics (ranging from 40 to 68) were likely a reflection of the diverse composition of the evaluation committee members across different geographies and transmission settings within SSA. Committee members remarked on these differences during the meetings convened to discuss the evaluation scores and rankings, reflecting that members perspectives of priority topics are in large part based on their background and country experience.

Several key themes emerged from this process. First, NMPs continue to face a multitude of challenges in the implementation of core malaria prevention and control interventions. The challenges identified by stakeholders are not new, but rather reflect persistent and intractable issues faced by NMPs and more broadly, ministries of health. Many of the challenges emphasized by stakeholders relate to broader health system issues—insufficient human and financial resources, inadequately trained workforce, supply chain failures leading to commodity stockouts, poorly integrated and inadequate data systems, and lack of high-quality data for decision-making—that act as barriers to NMPs in achieving their national malaria strategic plan goals and coverage targets. These challenges have been previously documented and recognized as critical to enhance progress in malaria control and elimination [24, 25]. It is important to recognize that some of these broad health systems challenges can be in part addressed through research but will also require broader policy and health system investments to overcome.

Another emergent theme was that NMPs feel that there is a lack of evidence on the effectiveness and cost-effectiveness of specific interventions and intervention packages to inform programmatic decision-making, especially with respect to sub-national tailoring and targeting of interventions. As new tools become available and are introduced at country level, this evidence will become even more critical given stagnant budgets. The perceived lack of sufficient evidence may be in part driven by the inadequate dissemination of research findings and translation of the evidence for policy and programmatic use, a challenge previously highlighted in many middle- and low-income countries [26–28]. This finding emphasizes the need for greater efforts in ensuring country stakeholder engagement in research from inception through to the dissemination and use of findings. Investment in capacity strengthening of policy and programme managers in the use of evidence to inform decision-making was highlighted as a critical gap to address [28].

Lastly, the findings reiterated the challenges that NMPs face in achieving the core intervention coverage targets outlined in their national malaria strategic plans. The prioritized OR and PE topics highlight the importance of identifying solutions to overcome the challenges of achieving and maintaining high coverage of core interventions, as well as improving the effectiveness of their delivery. While substantial progress has been made in the scale-up of interventions over the past two decades, gaps in coverage targets persist [1]. OR and PE will be a critical component to reducing these coverage gaps, along with broader policy and financial investment support.

A key objective for developing this prioritized list of OR and PE topics is to support and guide more coordinated investments in the research priorities by funding agencies. These agencies were actively engaged in the process from inception to garner their buy-in and help drive their use of the outputs to inform future investment decisions. It should be noted that the 33 research priorities identified in this process represent common themes and priorities rather than specific research questions. Funders can use this list as a starting point, and work with NMPs and their research partners to define contextually specific and relevant research questions. The outputs may also serve as a resource for NMPs and researchers from malaria-affected countries to advocate and position for funding on topics that align with their country research priorities. The list can also complement and supplement other processes undertaken at the country level to identify priority research questions. Additionally, as progress is made against these priorities, new research questions will inevitably emerge, highlighting the importance of regularly updating the priority list.

The process used for this research prioritization effort aimed to build upon other recent malaria research prioritization efforts. The desk review revealed a few country- and regional-level malaria research agendas that included some similarly themed operational research questions [29–32]. However, most of these agendas were set between 2015 and 2017 and were thus somewhat outdated. At the global level, the most recent research prioritization process was malERA Refresh in 2017 [8], which built upon the original malERA conducted in 2011 [11]. The malERA Refresh included some health systems and operational research-related questions but was largely focused on basic science and upstream research with limited country stakeholder involvement [6, 8, 12–17, 33]. What differentiates this prioritization process from previous efforts is an explicit focus on research to address operational challenges and evidence gaps as identified by country stakeholders, including NMPs and their research partners.

A strength of this process was that we adapted the CHNRI method [22], which provides a systematic framework and process for identifying, evaluating, and prioritizing research questions using a set of agreed upon evaluation criteria. The CHNRI method, initially developed in 2007, has since become the most commonly used methodology for prioritization of health research questions [23, 34]. The approach used for the stakeholder consultations was a unique adaptation of the CHNRI methodology; in the typical CHNRI process, experts are asked to share priority research questions on the specified topic area. During the consultations undertaken as part of our process, stakeholders were first asked to reflect on and discuss key programme implementation challenges and bottlenecks facing NMPs and what they perceived as the most pressing evidence gaps in malaria policy, strategy, and guidelines. This set of questions was then followed by asking stakeholders to identify priority OR and PE questions that could specifically address the identified challenges and gaps. This approach resulted in a set of OR and PE questions directly linked to addressing the identified challenges and gaps. Finally, the use of an independent expert evaluation committee provided further validation of the importance of the OR and PE questions identified and allowed for further refinement of the research topics to improve clarity.

This prioritization process had a few limitations. Although the process included input from a broad group of stakeholders within SSA, there was limited participation from stakeholders in lower transmission and elimination settings. As a result, the identified OR and PE priorities largely reflect issues in high and moderate malaria transmission settings. The robustness of the process was also impacted both by the timeframe for gathering stakeholder inputs and non-response among several invited stakeholders to provide inputs. It will be useful to explore other platforms or approaches to improve stakeholder participation for future iterations of the process and consider developing separate research agendas for high/moderate transmission settings and lower transmission and elimination settings within SSA.

## Conclusion

The research prioritization process was a valuable exercise to identify key operational challenges faced by NMPs, pressing evidence gaps, and a set of priority OR and PE topics for the SSA region. The prioritized list of topics can serve as an important resource to support funding agency alignment with country priorities and for furthering partnerships with national stakeholders toward formulating specific and relevant research questions for their country context. Ensuring sufficient investment to address the prioritized topics will be a critical step to addressing the persistent challenges and coverage gaps, and helping to reignite progress in malaria control and elimination. It will be important to track progress and regularly update this list to ensure its continued relevance.

## Supplementary Information

**Additional file 1: Table S1. **Evaluation criteria definitions. **Table S2.** Evaluation criteria scores for OR and PE topics by evaluation criteria question and overall research priority score. **Table S3.** Average expert agreement scores for OR and PE topics by criteria question and overall.

## Data Availability

Data gathered from the prioritization process will be made available from the corresponding authors upon reasonable request.

## Abbreviations

AEA: Average expert agreement
BMGF: Bill & Melinda Gates Foundation
CE: Community engagement
CHNRI: Child Health and Nutrition Research Initiative
FGD: Focus group discussion
GF: The Global Fund to Fight AIDS, Tuberculosis, and Malaria
HMIS: Health management information system
IPTp: Intermittent preventive treatment in pregnancy
IRS: Indoor residual spraying
ITN: Insecticide-treated nets
KII: Key informant interview
LSM: Larval source management
malERA: Malaria Eradication Research Agenda
MDA: Mass drug administration
NMP: National malaria programme
OR: Operational research
PE: Programme evaluation
PMC: Perennial malaria chemoprevention
PMI: U.S. President’s Malaria Initiative
RPS: Research priority score
SBC: Social and behaviour change
SMC: Seasonal malaria chemoprevention
SME: Surveillance, monitoring, and evaluation
SSA: Sub-Saharan Africa
UCAD: Université Cheikh Anta Diop of Dakar
WHO: World Health Organization
