# Supplementary material for: Defining operational research priorities to improve malaria control and elimination in sub-Saharan Africa: results from a country-driven research prioritization setting process
Source: Malar J. 2023 Jul 30;22:219. doi: 10.1186/s12936-023-04654-8 (PMC10387205; doi:10.1186/s12936-023-04654-8)
Supplement: Supplementary file 1 — Additional file 1: Table S1. Evaluation criteria definitions. Table S2. Evaluation criteria scores for OR and PE topics by evaluation criteria question and overall research priority score. Table S3. Average expert agreement scores for OR and PE topics by criteria question and overall. [file 12936_2023_4654_MOESM1_ESM.docx]

**Additional File**

## Evaluation Criteria Definitions

Table S1 presents the definitions for the six evaluation criteria used in the research prioritization setting process.

Table S1. Evaluation criteria definitions

| **No.** | **Criteria** | **Definition** |
| --- | --- | --- |
| 1 | Broad relevance | This criterion assesses the relevance of the research priority across a relatively large number of malaria-endemic settings. This criterion will take into consideration the potential relevance of the priority across multiple settings and countries, thus demonstrating that addressing the priority would inform the work of multiple country programmes. |
| 2 | High impact on malaria burden | This criterion assesses the extent to which the research priority would address significant coverage gaps, challenges, or barriers in the uptake of or access to malaria interventions among populations at risk, and therefore lead to a substantial impact on malaria burden relative to the specific country or subnational context. For high and moderate transmission settings, a research priority would be considered to have high impact if it would lead to optimization of a proven intervention and thus likely contribute to a substantial reduction in malaria mortality, incidence, and/or malaria prevalence. In low and very low transmission settings, leading to high impact is likely to be more nuanced and difficult to measure, and will largely be based on the context. In these settings it would be expected that addressing the research gap/priority would contribute to reducing threats to intervention effectiveness (e.g., mitigating threats to drug and insecticide resistance), strengthening the robustness of a country’s surveillance system, or bringing a setting(s) closer to and achieving elimination. |
| 3 | Improves efficiency | Assesses the extent to which the research priority will help improve efficiency in the delivery of an intervention or approach. In this context, improved efficiency could refer for example to the optimization of how an intervention is delivered in terms of reducing unnecessary resources or costs, changing how an intervention is delivered to improve the quality and/or its effectiveness, or improvements in data quality, accuracy, or timeliness, from data or digital innovations. Efficiency could also be conceptualized in terms of potentially withdrawing or replacing an intervention with another proven intervention to improve efficiency or cost-effectiveness. |
| 4 | Addresses inequities | Evaluates the extent to which the research priority will help address inequities in access to interventions and coverage gaps with an emphasis on gender, geographical (specifically, hard-to-reach populations), demographic (with a focus on high-risk and more vulnerable populations, such as children under five years of age and pregnant women), and socio-economic factors. |
| 5 | Scalability and sustainability | Assesses the extent to which the research priority addresses an approach or intervention that can be feasibly and effectively delivered at scale within countries’ existing systems to reach the eligible population(s) in need. Several aspects will be taken into consideration to assess scalability of an approach or intervention, such as the cost required, available resources and supporting systems within the country, ability of the approach/intervention to remain effective as it is scaled up, local ownership, political will, and sustainability. |
| 6 | Feasibility | Assesses whether the research priority is answerable using available research methods and in alignment with ethical principles (e.g., research does not raise any major ethical concerns). The criteria also assesses whether the research can be answered in an efficient manner, both in terms of the cost/affordability and timeliness for conducting the research (such that the research findings when available will still be relevant). |

## Methodology for calculated research priority scores

### Research Priority Score Calculation

The research priority score (RPS) represents the average score across the evaluators and evaluation criteria questions (*q*). The RPS was calculated for each research priority separately. A numeric score was assigned to the ranking the evaluator assigned for each of the 10 evaluation criteria scores: 1=strongly disagree; 2=disagree; 3=neither agree nor disagree; 4=agree; and 5=strongly agree. The specific calculation was as follows:

*RSP* = $\frac{\sum_{q=1}^{10} numeric score}{5*\sum_{q=1}^{10} number of respondents}$

A detailed explanation of the calculation of the RSP is provided below. For our paper, we expressed the RPS as a percentage.

Using the following definitions:

- BR1=broad relevance criteria question 1
- HI1=high impact criteria question 1
- HI2=high impact criteria question 2
- IE1=improves efficiency criteria question 1
- IE2=improves efficiency criteria question 2
- A11=addresses inequities criteria question 1
- AI2=addresses inequities criteria question 2
- SS1=scalability and sustainability criteria question 1
- SS2=scalability and sustainability criteria question 2
- F1=feasibility criteria question 1
- F2=feasibility criteria question 2
- *t*=total score
- *n*=number of evaluators/scorers

RPS = $\frac{tBR1+tH1+tH2+tIE1+tIE2+tAI1+tAI2+tSS1+tSS2+tF1+tF2}{5*(nBR1+nH1+nH2+nIE1+nIE2+nAI1+nAI2+nSS1+nSS2+nF1+nF2)}$ X 100

### Average expert agreement calculation

The average expert agreement (AEA) score represents the degree to which evaluators agree on the scores they provided. It was calculated for each evaluation criteria and then averaged across for an overall AEA score for each research priority. The AEA was expressed as a percentage. The AEA was calculated as follows:

AEA = $\frac{1}{10}$ $\sum_{q=1}^{10} \frac{Number of scorers who provided the modal response}{Number of scorers}$ X 100

## Detailed Research Priority Scores

Table S2. Evaluation criteria scores for OR and PE topics by evaluation criteria question and overall research priority score.

| **Operational Research/Programme Evaluation Topic** | **Evaluation Criteria** | | | | | | | | | | **RPS**  **Overall** |
| --- | --- | --- | --- | --- | --- | --- | --- | --- | --- | --- | --- |
|  | **BR** | **HI1** | **HI2** | **IE1** | **IE2** | **AI1** | **AI2** | **SS** | **F1** | **F2** |  |
| Test and evaluate different delivery mechanisms to reach and sustain high coverage of ITNs among hard-to-reach and highest risk populations | 88.2 | 88.2 | 85.9 | 88.2 | 85.9 | 94.1 | 90.6 | 85.9 | 87.1 | 84.7 | 87.9 |
| Evaluate the effectiveness and cost-effectiveness of different strategies for deploying the RTS, S AS01 malaria vaccine with chemoprevention (e.g., campaign vs. EPI-linked vs combination campaign/EPI strategies). | 91.8 | 90.0 | 86.3 | 90.6 | 88.2 | 88.2 | 84.7 | 83.5 | 83.5 | 78.8 | 86.6 |
| Assess the effectiveness and cost-effectiveness of different intervention combinations (e.g., ITNs + IRS, ITNs or IRS + LSM, vector control + chemoprevention) to better understand how interventions should be combined to maximize impact. | 94.1 | 84.7 | 85.9 | 90.6 | 87.5 | 77.5 | 76.5 | 88.2 | 84.7 | 83.5 | 85.3 |
| Test and evaluate approaches or interventions to reduce the frequency of stockouts of key commodities for malaria case management, especially at the community level. | 85.9 | 85.9 | 83.8 | 84.7 | 88.2 | 85.9 | 87.1 | 83.5 | 82.4 | 85.9 | 85.3 |
| Evaluate and compare different insecticide management and/or rotation strategies on insecticide resistance prevalence and intensity. | 94.1 | 77.6 | 82.4 | 85.9 | 91.8 | 81.2 | 78.8 | 87.1 | 85.9 | 85.9 | 85.1 |
| Evaluate the impact and cost-effectiveness of expanding the age range, geographical coverage, and rounds of treatment of seasonal malaria chemoprevention. | 89.4 | 84.7 | 90.6 | 77.6 | 80.0 | 87.1 | 82.4 | 84.7 | 85.9 | 82.4 | 84.5 |
| Assess factors associated with CHW motivation and retention and evaluate different approaches or interventions to improve CHW motivation and retention. | 85.9 | 84.7 | 75.3 | 75.3 | 81.2 | 88.2 | 85.9 | 81.2 | 88.2 | 87.1 | 83.3 |
| Assess predictors of adherence to and determinants of uptake of SMC and evaluate different strategies to achieving high SMC coverage and adherence. | 80.0 | 80.0 | 78.8 | 82.4 | 84.7 | 88.2 | 82.4 | 81.3 | 82.5 | 82.7 | 82.3 |
| Test and evaluate the effectiveness of different deployment and targeting approaches for IRS to maximize impact (e.g., testing different insecticides, duration and frequency of spraying, geographic/structural targeting strategies). | 85.9 | 80.0 | 82.4 | 87.1 | 87.1 | 75.3 | 77.6 | 81.2 | 81.2 | 82.4 | 82.0 |
| Assess different approaches or interventions to improve the analytic and data use capacity, and data use culture at different levels of the health system. | 81.2 | 75.3 | 72.9 | 83.5 | 83.5 | 76.5 | 82.4 | 82.4 | 88.2 | 87.1 | 81.3 |
| Assess the impact of IRS and focal/reactive IRS on malaria burden, transmission, and insecticide resistance. | 83.5 | 77.6 | 82.4 | 84.7 | 87.1 | 76.5 | 75.3 | 81.2 | 83.5 | 81.2 | 81.3 |
| Given the challenges with ITN durability, test and evaluate the effectiveness of different approaches to improve routine/continuous distribution channels for ITNs to sustain coverage between mass campaigns. | 84.7 | 85.9 | 74.1 | 84.7 | 82.4 | 78.8 | 77.5 | 82.4 | 81.2 | 81.2 | 81.3 |
| Compare different SBC/community engagement strategies in terms of effectiveness and cost-effectiveness on healthcare seeking, adherence to treatment, and uptake of key prevention interventions. | 82.4 | 82.4 | 80.0 | 84.7 | 83.5 | 81.2 | 80.0 | 80.0 | 78.8 | 76.3 | 80.9 |
| Assess the effectiveness and cost-effectiveness of innovative approaches to reduce the cost and/or improve the efficiency of IRS implementation (e.g., partial spraying of structures, use of a decentralized approach, targeted spraying). | 85.9 | 81.2 | 77.6 | 88.2 | 80.0 | 75.3 | 78.8 | 77.6 | 83.5 | 80.0 | 80.8 |
| Assess structural and behavioural factors associated with delayed care-seeking across different population groups (e.g., age, gender, hard-to-reach/vulnerable populations) and compare different strategies to decrease delays in care-seeking. | 81.2 | 84.7 | 78.8 | 76.5 | 75.3 | 84.7 | 83.5 | 76.5 | 80.0 | 78.8 | 80.0 |
| Assess predictors of adherence and non-adherence to case management treatment guidelines among health care providers and test/evaluate different strategies to improve adherence to guidelines. | 83.5 | 82.4 | 77.5 | 78.8 | 84.7 | 75.3 | 70.6 | 77.6 | 82.4 | 82.4 | 79.5 |
| Evaluate how current surveillance systems are functioning, and whether they are producing reliable and accurate information to guide countries toward elimination. | 88.2 | 80.0 | 74.1 | 78.8 | 72.9 | 76.5 | 77.6 | 78.8 | 82.4 | 84.7 | 79.4 |
| Assess the operational feasibility and most effective delivery platform for perennial malaria chemoprevention administration (e.g., EPI, mass campaign, community health workers). | 81.2 | 75.0 | 69.4 | 87.1 | 76.5 | 81.2 | 77.5 | 80.0 | 82.4 | 78.8 | 78.9 |
| Assess the feasibility and benefit of different digital tools/systems for use at the community level for data capture, reporting, and transmission to HMIS/DHIS2. | 87.1 | 72.9 | 68.2 | 77.6 | 80.0 | 74.1 | 75.3 | 78.8 | 85.9 | 87.1 | 78.7 |
| Evaluate different strategies for achieving high MDA coverage and adherence in different transmission contexts. | 85.0 | 81.3 | 76.3 | 81.3 | 78.8 | 72.5 | 73.8 | 81.3 | 80.0 | 76.0 | 78.6 |
| Test and evaluate interventions to improve adherence to malaria treatment guidelines and reporting in private sector health facilities. | 82.4 | 77.6 | 78.8 | 77.6 | 82.4 | 70.6 | 75.3 | 75.3 | 81.2 | 84.7 | 78.6 |
| Assess the long-term effectiveness and sustainability of different social and behaviour change approaches on key malaria treatment and prevention behaviours and the duration of their impact on intervention uptake. | 83.5 | 82.4 | 75.0 | 77.6 | 81.2 | 74.1 | 76.5 | 74.1 | 75.3 | 81.2 | 78.1 |
| Compare different strategies for surveillance and response in elimination settings, assessing completeness, timeliness, delivery of response, and cost-effectiveness. | 80.0 | 74.1 | 77.6 | 81.2 | 84.7 | 71.8 | 71.8 | 76.5 | 78.8 | 83.8 | 78.0 |
| Test the effectiveness of different strategies to improve early ANC attendance and IPTp coverage. | 78.8 | 81.2 | 78.8 | 78.8 | 80.0 | 80.0 | 78.8 | 76.5 | 77.6 | 77.6 | 78.0 |
| Test and evaluate strategies to improve the efficiency of the delivery of IPTp (e.g., community-based delivery through community health workers)? | 80.0 | 81.2 | 68.2 | 80.0 | 78.8 | 80.0 | 80.0 | 69.4 | 80.0 | 81.2 | 77.9 |
| Test and evaluate different approaches or interventions for improving HMIS data quality. | 84.7 | 75.3 | 69.4 | 76.5 | 76.5 | 72.9 | 75.3 | 81.2 | 83.5 | 81.2 | 77.6 |
| Evaluate different strategies to improve health care worker adherence to IMCI guidelines. | 78.8 | 76.3 | 73.8 | 81.3 | 76.3 | 80.0 | 75.0 | 76.3 | 75.0 | 81.3 | 77.4 |
| Evaluate the effectiveness and cost-effectiveness of larval source management on epidemiological and entomological outcomes in different transmission contexts and the duration of impact. | 88.2 | 75.3 | 74.1 | 75.3 | 82.4 | 71.8 | 68.2 | 70.6 | 81.2 | 78.8 | 76.6 |
| Test approaches or strategies to improve cost and resource efficiency (e.g., integration of seasonal malaria chemoprevention with other delivery platforms) and to maintain effectiveness in the delivery of seasonal malaria chemoprevention when scaling up the intervention. | 77.6 | 77.6 | 76.5 | 81.2 | 77.6 | 77.6 | 74.1 | 74.1 | 72.9 | 76.5 | 76.6 |
| Compare or evaluate different strategies/packages of interventions to maintain low/current malaria case incidence following the withdrawal of IRS. | 88.2 | 68.2 | 75.3 | 78.8 | 76.5 | 72.9 | 72.9 | 80.0 | 77.6 | 75.3 | 76.6 |
| Assess barriers and facilitators to ITN use in different settings where access to ITNs is high and evaluate the effectiveness of different social and behaviour change (SBC) approaches/interventions to improve ITN use within different settings/contexts based on the identified barriers. | 80.0 | 76.5 | 73.8 | 76.5 | 78.8 | 77.6 | 76.5 | 75.3 | 70.6 | 77.6 | 76.3 |
| Test different approaches for working with/incentivizing participation and collaboration of the private sector in the referral, diagnosis, treatment, and reporting of malaria cases. | 85.9 | 78.8 | 72.9 | 75.3 | 81.2 | 69.4 | 67.1 | 70.6 | 76.5 | 77.6 | 75.5 |
| Assess the magnitude of cross border movement of people on malaria incidence/prevalence and evaluate the effectiveness of different strategies to reduce malaria transmission along international borders. | 82.4 | 72.9 | 68.2 | 65.0 | 68.2 | 72.9 | 72.9 | 64.7 | 75.0 | 72.5 | 71.5 |

Notes: BR=broad relevance criteria; HI1=high impact criteria question 1; H12=high impact criteria question 2; IE1=improves efficiency criteria question 1; IE2=improves efficiency criteria question 2; AI1=addresses inequities criteria question 1; AI2=addresses inequities criteria question 2; SS=sustainability and scalability criteria; F1=feasibility criteria question 1; F2=feasibility criteria question 2.

Table S3. Average expert agreement scores for OR and PE topics by criteria question and overall

| **Operational Research/Programme Evaluation Topic** | **Evaluation Criteria** | | | | | | | | | | **AEA Overall** |
| --- | --- | --- | --- | --- | --- | --- | --- | --- | --- | --- | --- |
|  | **BR** | **HI1** | **HI2** | **IE1** | **IE2** | **AI1** | **AI2** | **SS** | **F1** | **F2** |  |
| Test approaches or strategies to improve cost and resource efficiency (e.g., integration of SMC with other delivery platforms) and to maintain effectiveness in the delivery of seasonal malaria chemoprevention when scaling up the intervention. | 70.6 | 76.5 | 58.8 | 58.8 | 58.8 | 52.9 | 70.6 | 70.6 | 76.5 | 82.4 | 67.6 |
| Assess the long-term effectiveness and sustainability of different social and behaviour change approaches on key malaria treatment and prevention behaviours and the duration of their impact on intervention uptake. | 52.9 | 58.8 | 50.0 | 58.8 | 58.8 | 58.8 | 70.6 | 58.8 | 70.6 | 70.6 | 60.9 |
| Given the challenges with ITN durability, test and evaluate the effectiveness of different approaches to improve routine/continuous distribution channels for ITNs to sustain coverage between mass campaigns. | 52.9 | 58.8 | 35.3 | 64.7 | 64.7 | 64.7 | 68.8 | 64.7 | 64.7 | 64.7 | 60.4 |
| Test and evaluate different delivery mechanisms to reach and sustain high coverage of ITNs among hard-to-reach and highest risk populations. | 47.1 | 58.8 | 58.8 | 58.8 | 58.8 | 70.6 | 52.9 | 58.8 | 64.7 | 64.7 | 59.4 |
| Test the effectiveness of different strategies to improve early ANC attendance and IPTp coverage. | 47.1 | 64.7 | 64.7 | 64.7 | 76.5 | 58.8 | 70.6 | 52.9 | 52.9 | 58.8 | 58.2 |
| Evaluate different strategies to improve health care worker adherence to IMCI guidelines. | 68.8 | 43.8 | 37.5 | 68.8 | 75.0 | 50.0 | 43.8 | 50.0 | 62.5 | 81.3 | 58.1 |
| Test and evaluate different approaches or interventions for improving HMIS data quality. | 52.9 | 52.9 | 47.1 | 47.1 | 58.8 | 41.2 | 47.1 | 70.6 | 82.4 | 70.6 | 57.1 |
| Assess the effectiveness and cost-effectiveness of innovative approaches to reduce the cost and/or improve the efficiency of IRS implementation (e.g., partial spraying of structures, use of a decentralized approach, targeted spraying). | 47.1 | 41.2 | 52.9 | 47.1 | 52.9 | 58.8 | 64.7 | 58.8 | 64.7 | 70.6 | 55.9 |
| Compare different strategies for surveillance and response in elimination settings, assessing completeness, timeliness, delivery of response, and cost-effectiveness. | 52.9 | 41.2 | 58.8 | 58.8 | 64.7 | 41.2 | 35.3 | 58.8 | 75.0 | 68.8 | 55.6 |
| Test and evaluate interventions to improve adherence to malaria treatment guidelines and reporting in private sector health facilities. | 64.7 | 41.2 | 47.1 | 70.6 | 64.7 | 41.2 | 47.1 | 64.7 | 58.8 | 52.9 | 55.3 |
| Evaluate the impact and cost-effectiveness of expanding the age range, geographical coverage, and rounds of treatment of seasonal malaria chemoprevention. | 58.8 | 47.1 | 58.8 | 70.6 | 52.9 | 52.9 | 64.7 | 52.9 | 47.1 | 47.1 | 55.3 |
| Compare different SBC/community engagement strategies in terms of effectiveness and cost-effectiveness on healthcare seeking, adherence to treatment, and uptake of key prevention interventions. | 52.9 | 64.7 | 35.3 | 41.2 | 58.8 | 47.1 | 41.2 | 76.5 | 58.8 | 75.0 | 55.1 |
| Assess structural and behavioural factors associated with delayed care-seeking across different population groups (e.g., age, gender, hard-to-reach/vulnerable populations) and compare different strategies to decrease delays in care-seeking. | 58.8 | 47.1 | 64.7 | 41.2 | 41.2 | 41.2 | 58.8 | 58.8 | 70.6 | 64.7 | 54.7 |
| Evaluate and compare different insecticide management and/or rotation strategies on insecticide resistance prevalence and intensity (crosscuts use of ITNs and IRS). | 70.6 | 47.1 | 41.2 | 47.1 | 58.8 | 58.8 | 47.1 | 64.7 | 52.9 | 52.9 | 54.1 |
| Test different approaches for working with/incentivizing participation and collaboration of the private sector in the referral, diagnosis, treatment, and reporting of malaria cases. | 47.1 | 58.8 | 47.1 | 47.1 | 47.1 | 47.1 | 35.3 | 52.9 | 82.4 | 70.6 | 53.5 |
| Assess the effectiveness and cost-effectiveness of different intervention combinations (e.g., ITNs + IRS, ITNs or IRS + LSM, vector control + chemoprevention) to better understand how interventions should be combined to maximize impact. | 76.5 | 64.7 | 52.9 | 58.8 | 50.0 | 50.0 | 41.2 | 47.1 | 47.1 | 47.1 | 53.5 |
| Evaluate the effectiveness and cost-effectiveness of different strategies for deploying the RTS, S AS01 malaria vaccine with chemoprevention (e.g., campaign vs. EPI-linked vs combination campaign/EPI strategies). | 64.7 | 56.3 | 50.0 | 58.8 | 52.9 | 52.9 | 47.1 | 47.1 | 58.8 | 41.2 | 53.0 |
| Assess predictors of adherence to and determinants of uptake of SMC and evaluate different strategies to achieving high SMC coverage and adherence. | 58.8 | 41.2 | 47.1 | 41.2 | 52.9 | 47.1 | 64.7 | 62.5 | 50.0 | 60.0 | 52.5 |
| Assess the impact of IRS and focal/reactive IRS on malaria burden, transmission, and insecticide resistance. | 47.1 | 52.9 | 52.9 | 47.1 | 64.7 | 35.3 | 41.2 | 47.1 | 70.6 | 64.7 | 52.4 |
| Evaluate the effectiveness and cost-effectiveness of larval source management on epidemiological and entomological outcomes in different transmission contexts and the duration of impact. | 52.9 | 47.1 | 35.3 | 41.2 | 64.7 | 52.9 | 58.8 | 41.2 | 58.8 | 70.6 | 52.4 |
| Test and evaluate the effectiveness of different deployment and targeting approaches for IRS to maximize impact (e.g., testing different insecticides, duration and frequency of spraying, geographic/structural targeting strategies). | 52.9 | 47.1 | 41.2 | 47.1 | 64.7 | 47.1 | 35.3 | 47.1 | 64.7 | 58.8 | 50.6 |
| Evaluate how current surveillance systems are functioning, and whether they are producing reliable and accurate information to guide countries toward elimination. | 47.1 | 47.1 | 35.3 | 52.9 | 52.9 | 41.2 | 35.3 | 47.1 | 64.7 | 58.8 | 48.2 |
| Test and evaluate approaches or interventions to reduce the frequency of stockouts of key commodities for malaria case management, especially at the community level. | 47.1 | 47.1 | 50.0 | 41.2 | 52.9 | 47.1 | 47.1 | 52.9 | 47.1 | 47.1 | 47.9 |
| Assess factors associated with CHW motivation and retention and evaluate different approaches or interventions to improve CHW motivation and retention. | 52.9 | 47.1 | 35.3 | 29.4 | 70.6 | 58.8 | 47.1 | 41.2 | 47.1 | 47.1 | 47.6 |
| Evaluate different strategies for achieving high MDA coverage and adherence in different transmission contexts. | 43.8 | 43.8 | 50.0 | 37.5 | 43.8 | 37.5 | 37.5 | 68.8 | 68.8 | 40.0 | 47.1 |
| Compare or evaluate different strategies/packages of interventions to maintain low/current malaria case incidence following the withdrawal of IRS. | 52.9 | 35.3 | 47.1 | 41.2 | 35.3 | 35.3 | 41.2 | 70.6 | 58.8 | 52.9 | 47.1 |
| Test and evaluate strategies to improve the efficiency of the delivery of IPTp (e.g., community-based delivery through community health workers)? | 41.2 | 41.2 | 35.3 | 58.8 | 52.9 | 47.1 | 47.1 | 35.3 | 56.3 | 52.9 | 46.8 |
| Assess predictors of adherence and non-adherence to case management treatment guidelines among health care providers and test/evaluate different strategies to improve adherence to guidelines. | 41.2 | 41.2 | 31.3 | 52.9 | 41.2 | 58.8 | 47.1 | 41.2 | 52.9 | 52.9 | 46.1 |
| Assess different approaches or interventions to improve the analytic and data use capacity, and data use culture at different levels of the health system. | 52.9 | 35.3 | 29.4 | 41.2 | 47.1 | 47.1 | 41.2 | 47.1 | 58.8 | 52.9 | 45.3 |
| Assess the feasibility and benefit of different digital tools/systems for use at the community level for data capture, reporting, and transmission to HMIS/DHIS2. | 47.1 | 41.2 | 29.4 | 47.1 | 52.9 | 29.4 | 29.4 | 41.2 | 70.6 | 64.7 | 45.3 |
| Assess the magnitude of cross border movement of people on malaria incidence/prevalence and evaluate the effectiveness of different strategies to reduce malaria transmission along international borders. | 41.2 | 41.2 | 29.4 | 37.5 | 47.1 | 47.1 | 52.9 | 35.3 | 56.3 | 62.5 | 45.0 |
| Assess the operational feasibility and most effective delivery platform for perennial malaria chemoprevention administration (e.g., EPI, mass campaign, community health workers). | 52.9 | 37.5 | 35.3 | 52.9 | 35.3 | 41.2 | 31.3 | 35.3 | 52.9 | 52.9 | 42.8 |
| Assess barriers and facilitators to ITN use in different settings where access to ITNs is high and evaluate the effectiveness of different social and behaviour change (SBC) approaches/interventions to improve ITN use within different settings/contexts based on the identified barriers. | 47.1 | 35.3 | 43.8 | 41.2 | 35.3 | 41.2 | 35.3 | 52.9 | 29.4 | 41.2 | 40.3 |

Notes: BR=broad relevance criteria; HI1=high impact criteria question 1; H12=high impact criteria question 2; IE1=improves efficiency criteria question 1; IE2=improves efficiency criteria question 2; AI1=addresses inequities criteria question 1; AI2=addresses inequities criteria question 2; SS=sustainability and scalability criteria; F1=feasibility criteria question 1; F2=feasibility criteria question 2.
